# Therapeutic targeting of leukemia stem cells in acute myeloid leukemia

**DOI:** 10.3389/fonc.2023.1204895

**Published:** 2023-08-03

**Authors:** Karina Barbosa, Aniruddha J. Deshpande

**Affiliations:** Tumor Initiation and Maintenance Program, Sanford Burnham Prebys Medical Discovery Institute, La Jolla, CA, United States

**Keywords:** cancer stem cells, leukemia stem cells (LSCs), acute myeloid leukemia, self-renewal and differentiation, malignant hematopoiesis

## Abstract

One of the distinguishing properties of hematopoietic stem cells is their ability to self-renew. Since self-renewal is important for the continuous replenishment of the hematopoietic stem cell pool, this property is often hijacked in blood cancers. Acute myeloid leukemia (AML) is believed to be arranged in a hierarchy, with self-renewing leukemia stem cells (LSCs) giving rise to the bulk tumor. Some of the earliest characterizations of LSCs were made in seminal studies that assessed the ability of prospectively isolated candidate AML stem cells to repopulate the entire heterogeneity of the tumor in mice. Further studies indicated that LSCs may be responsible for chemotherapy resistance and therefore act as a reservoir for secondary disease and leukemia relapse. In recent years, a number of studies have helped illuminate the complexity of clonality in bone marrow pathologies, including leukemias. Many features distinguishing LSCs from normal hematopoietic stem cells have been identified, and these studies have opened up diverse avenues for targeting LSCs, with an impact on the clinical management of AML patients. This review will discuss the role of self-renewal in AML and its implications, distinguishing characteristics between normal and leukemia stem cells, and opportunities for therapeutic targeting of AML LSCs.

## AML as a stem cell disease

Hematopoiesis is organized as a hierarchy, with hematopoietic stem cells (HSCs) at the apex. Blood homeostasis relies on the regulated differentiation of HSCs into the diverse cell types that constitute blood. In normal hematopoiesis, blood production is sustained by HSCs, which create committed progenitor cells that multiply and differentiate into functional blood cells of distinct lineages (1). Central to this process is the self-renewing capacity of HSCs, which ensures that the pool of uncommitted stem cells is not exhausted. Importantly, HSCs are capable of self-renewal, but upon differentiation, their progeny are severely limited in this capacity (1).

**Figure 1:**
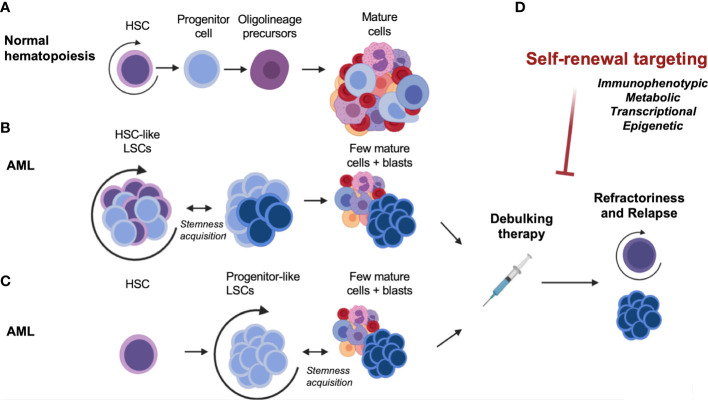
Self-renewal, an essential component of normal and malignant hematopoiesis. **(A)** Normal hematopoiesis. **(B)** Malignant hematopoiesis sustained by HSC-like LSCs. **(C)** Malignant hematopoiesis sustained by progenitor-like LSCs. **(D)** Therapeutic targeting and disease relapse. AML, acute myeloid leukemia; HSC, hematopoietic stem cell; LSC, leukemia stem cell.

In Acute Myeloid Leukemia (AML), hematopoietic stem or progenitor cells acquire genetic alterations that confer pre-leukemic features of heightened competitive fitness, including proliferative expansion and enhanced survival (2). The cellular heterogeneity of AML is believed to reflect the hierarchical organization of normal hematopoietic differentiation, with undifferentiated stem-like cells termed leukemia stem cells (LSCs) at the apex of the hierarchy giving rise to more differentiated AML cells (**Figure 1A**). In a seminal paper, the biological diversity and organization of the leukemic clone within AML patients were demonstrated by prospective isolation of immunophenotypically defined populations, followed by engraftment studies in non-obese diabetic/severe combined immunodeficient (NOD/SCID) mice (3). The authors showed that highly purified CD34+ CD38- cells but not CD34- or CD34+/CD38+ cells from AML patient-derived xenograft (PDX) samples could efficiently repopulate AML in mice. Importantly, this model implied that only the LSC fraction within the tumor was capable of reconstituting disease. These pioneering studies in the hematopoietic system paved the way for the identification and characterization of cancer stem cells in several other human cancers and helped establish the paradigm of the cancer stem cell (reviewed in (4)). Given the inherent limitations of *in vivo* xenotransplantation assays alternative techniques to study human AML-LSCs were also developed. For example, an *ex-vivo* bone marrow stromal co-culture system using CD34+ cells from peripheral blood samples from AML patients allowed long-term cultures to be maintained over 20 weeks (5). This assay model allowed a more detailed study of the interactions between LSCs and stromal cells on the bone marrow microenvironment, compared to *in vivo* settings. Based on these studies, LSCs were thought to divide in a manner analogous to benign HSCs, giving rise to a bulk population of AML blasts, while also retaining some biological properties of stem cells. Specifically, these include the propensity to extensively self-renew, alternate between dormant and cycling states, and exhibit resistance to cytotoxic therapies (2, 3, 6). However, subsequent studies have demonstrated that LSCs may also harbor complex features that distinguish them from normal HSCs. The similarities and distinctive features of HSCs and LSCs are of clinical relevance, since they may help determine a potential therapeutic window for AML-LSC targeting. These will be elaborated upon in the following section.

## Targeting of AML LSCs: exploiting the similarities and differences between HSCs and AML-LSCs

### Immunophenotype-based targeting of AML-LSCs

Several groups have sought to investigate the overlapping and distinct properties of normal HSCs and AML-LSCs in order to understand the means by which normal stemness attributes are co-opted in AML. In human AML, several studies showed that LSCs can share certain cell-surface markers that are used to define normal HSCs, including non-expression of lineage (Lin) markers, expression of the CD34 surface marker, and a CD38 negative (CD38-) immunophenotype. Later studies using immunodeficient mice – which are more permissive for engraftment of human cells – showed that human AML-LSCs from most patients reside in the CD34+/CD38- or CD34+/CD38+ population, and in a few AMLs such as NPM1 mutant AML, may also be present in the CD34- population (7, 8). Therefore, immunophenotypically, AML-LSCs may not entirely resemble normal human HSCs, but more of a multipotent progenitor (MPP) (9) or a granulocyte-macrophage progenitor (GMP)-like stage (10) (**Figures 1B, C**). In fact, a study on several human AML patient samples indicated that most AML-LSCs may reflect the reacquisition of stem cell characteristics in progenitor cells instead of actual HSCs (11). These results are consistent with findings from mouse models which sought to directly identify the LSC potential of different purified hematopoietic populations by introducing oncogenes such as MLL-AF9, HOXA9/MEIS1, MOZ-TIF2 or MN1 (10, 12–15). Through the use of these murine bone marrow transplantation models, it was shown that the expression of leukemia-inducing oncogenes, such as MLL-AF9, can activate stem cell-like gene expression programs in downstream progenitors such as GMPs (10). It is evident that even if the original oncogenic mutation emerges in HSCs, the LSC population –as defined by the population of cells that can regenerate the heterogeneity of the tumor in an experimental setting in mice – may still reside in a more downstream MPP- or GMP- like stage that aberrantly acquires self-renewal properties. Taken together, the mouse and human studies demonstrate that AML-LSCs can be immunophenotypically diverse compared to what was initially defined and may resemble more committed progenitor cells. Moreover, it has now become clear that the immunophenotype of AML-LSCs can also vary from patient-to-patient and from diagnosis to relapse (16–19). Thus, AML-LSCs can immunophenotypically resemble a spectrum of hematopoietic stem and progenitor cells, which has important ramifications for their surface-marker-based identification and therapeutic targeting.

Several different surface markers have been shown to be enriched in the LSC compartment including CD96 (20), CD123 (IL3Rα) (21), CD44 (22), CD47 (23), CLECL12A (CLL-1) (24) and GPR56 (25), although the degree to which their expression differs from normal HSCs varies greatly. Concerns derived from their shared expression with HSCs and ensuing toxicity have prompted careful evaluation of agents targeting these pathways or considering their use in combination with other drugs (26–29). A number of different strategies are being deployed for the therapeutic targeting of surface proteins in AML. These include unconjugated, or bi and tri-specific antibodies which can facilitate the engagement of T or NK cells against AML targets. Another approach being tested is the use of antibody-drug conjugates (ADCs) where selective antibodies are conjugated to a toxin (payload). Similarly, radio-conjugated antibodies can also enhance the cytotoxic potency of antibody-based therapies and are being tested in AML. Finally, the use of checkpoint inhibitors or chimeric antigen receptor T-cells (CAR-Ts) are other strategies that could be directed for LSC elimination as discussed below:

Some of the most common surface-associated antigens that have been pursued in clinical trials are CD33, FLT3 and CD123 (IL3-Ra). In each of these cases, trials have been discontinued due to unforeseen toxicities, raising questions about the selectivity of these surface markers on AML cells and particularly on AML-LSCs. Despite substantial toxicities, the ADC gentuzumab ozogamicin (GO) - a CD33 antibody conjugated to the potent enediyne DNA-binding cytotoxic antibiotic calicheamicin - is approved by the Food and Drug Administration for treating certain subtypes of AML. In core-binding factor AML, GO treatment in induction therapy increased long-term survival rates (30). Another anti-CD33-based ADC, vadastuxumab, which is based on the potent DNA minor groove-binding agent pyrrolobenzodiazepine, showed promise in preclinical models of AML, but the trial was discontinued due to safety issues (NCT02785900). Thus, therapies targeting CD33 have met with limited success. Similarly, clinical targeting of the CD123 surface maker (IL3Ra) has shown mixed results. A number of clinical trials with CD123-directed antibody therapies have been conducted, and while the results of some of the trials are still awaited, other trials had to be discontinued because of limited clinical efficacy, suboptimal drug exposure, or unfavorable safety profiles; e.g.: NCT02992860 (31). Recently, a Phase 1b/2 trial of IMGN632, a CD123-targeting antibody conjugated to an alkyl-benzodiazepine together with azacytidine and venetoclax (Aza/Ven) showed tolerable safety profiles and compelling preliminary anti-leukemia activity (NCT04086264) (32). Thus, CD123-based targeting may show promise in some patients with high CD123 expression, although the effect of these ADCs on normal HSCs remains to be seen given the importance of the IL3 signaling pathway in normal hematopoiesis (33). Currently, CD70-targeting antibodies (cusatuzumab), in combination with azaciditine or venetoclax, remain under clinical investigation with promising findings (34).

In addition to the use of antibody-based targeting approaches, there are also several ongoing efforts to engage T-cells using bispecific T-cell engaging antibodies (BiTEs), which recruit CD3 effector T cells to target tumor cells. Some of the early BiTEs tested in AML included AMG-330 AMG673, and AMV564, all of which are dual-targeting agents for CD33 and CD3. Each of these BiTEs showed early evidence of anti-leukemia activity in patients with relapsed/refractory AML (35, 36). Similarly, phase I dose-escalation trials with the CD123-CD3 targeting BiTE XmAb14045 and the dual-afffity retargeting (DART) antibody flotetuzumab showed promising anti-leukemia activity in heavily treated relapsed refractory AML patients, with manageable toxicities.

Aside from ADCs and BiTEs, CAR-T cells targeting CD33 and CD123 are also under clinical investigation in AML. Because of HSPC toxicity evident from early clinical studies, clinical trials are focusing on their therapeutic use as bridge-to-transplant regimes prior to allogeneic hematopoietic stem cell transplants (37, 38). Regarding checkpoint immunotherapy, the CD47 macrophage “don’t eat me” signal is upregulated in AML-LSCs and associated with poor prognosis (23, 39, 40). The monoclonal humanized antibody magrolimab binds CD47 and blocks its interaction with its ligand SIRPα in phagocytic cells, leading to the phagocytosis of AML cells. Recently, trials with Magrolinab and azacytidine combination in *de novo* AML patients showed durable efficacy in AML, including in p53-mutant AML, which is one of the most treatment-resistant subtypes of AML (41).

Thus, several types of immunophenotype-based strategies are under consideration for AML (**Table 1**), with some showing impressive clinical activity - although their effects on LSCs and on LSC-mediated AML relapse remain to be seen.

**Table 1:** Selected agents in clinical trials for AML as of June 2023.

Modality	Target	Drug	Trial Number
ADC	CD33	Gemtuzumab Ozogamicin	NCT03531918, NCT00658814
	ROR1	Zilovertamab vedotin	NCT03833180
	TN-C	F16IL2	NCT02957032
CAR-T cells	CD123	CAR-T cells	NCT02159495
Checkpoint inhibitor	CD47	Evorpacept (ALX148)	NCT04755244
		Lemzoparlimab	NCT04202003
	CD70	Cusatuzumab	NCT04150887, NCT04023526
	CTLA-4	Ipilimumab	NCT02890329
	PD-1	Nivolumab	NCT02275533, NCT02532231, NCT03825367, NCT03092674, NCT04277442
		Pembrolizumab	NCT02768792, NCT03761914
	PD-1 + CTLA-4	Nivolumab + Ipilimumab	NCT02846376
	PD-1 + TIM-3	MBG453 + PDR001	NCT03066648
	PD-L1	Atezolizumab	NCT02935361
	TIM3	MBG453	NCT03940352, NCT04150029
		SHR-1702	NCT04443751
DART	CD123	Flotetuzumab	NCT04582864, NCT04158739
Radio-immuno-conjugate	CD45	Iomab-B	NCT02665065
	CD33	Lintuzumab-Ac225	NCT03441048
Small molecule	ALDH	ABD-3001	NCT05601726
	E-selectin	Uproleselan	NCT03616470
	IDH1	FT-2102	NCT02719574
		IDH305	NCT02381886
		Ivosidenib	NCT03173248, NCT02632708
	IDH1/2	Ivosidenib, enasidenib	NCT02677922
		LY3410738	NCT04603001
	IDH2	Enasidenib	NCT03728335, NCT03515512, NCT03720366, NCT02577406, NCT04092179, NCT01915498
	MCL1	S64315	NCT04629443
	MEN1	DS-1594b	NCT04752163
	WNT	CWP232291	NCT03055286

DART, Dual-affinity retargeting antibody; ADC, Antibody-drug conjugate.

### Transcriptional states of self-renewal and differentiation

Early studies in the hematopoietic system helped define the property of stem cell self-renewal, which allows HSCs to produce mature blood cells while also retaining the undifferentiated stem cell pool (42). Similar to HSCs, AML-LSCs are also thought to be long-lived due to an enhanced replicative capacity as determined by long-term engraftment in immunocompromised mice (3, 43). Pioneering experiments first demonstrated long-term reconstitution of multi-lineage hematopoiesis in immunodeficient mice co-implanted with fragments of human fetal thymus and fetal liver (44), as well as unfractionated bone marrow (45). Leukemic cell engraftment in mice was first obtained with bulk AML samples (43) and later with purified subsets of AML cells, demonstrating the immunophenotypic origin of leukemia-initiating cells (3). The latter study also demonstrated two current tenets of the cancer stem cell model: that LSCs were able to proliferate and differentiate into blast populations immunophenotypically identical to those present in the original AML samples and that they were capable of extensive self-renewal *in vivo*, as measured by leukemia propagation in primary as well as secondary recipients. Collectively, these studies led to the hypothesis that, in the majority of cases, AML originates from -and is maintained by – transformed stem cells or by cells that have reacquired stem-cell attributes following transformation (8). When HSCs divide, they can either do so symmetrically, giving rise to two daughter stem cells or two daughter committed progenitor cells, or asymmetrically – giving rise to one stem cell and one differentiated or committed progenitor cell. When at least one of the daughter cells retains the property of self-renewal, the stem cell pool is replenished (42). Similarly, AML stem cells must be constantly replenished through the regeneration of AML-LSCs, achieved by the retention or re-initiation of self-renewal properties in AML cells. In addition, AML-LSCs also differentiate into the bulk of the tumor that recapitulates the cellular heterogeneity of the original disease, consisting mostly of AML blasts that have a much more limited self-renewing capability. However, a remarkable difference in comparison to HSCs is that AML-LSCs are more constrained in their differentiation potential (46, 47). This is largely due to perturbations in lineage-specifying transcription factors. An example is the myeloid transcription factor CEBPA, which is mutated in approximately 10 percent of AML. Other instances include the altered expression or inactivation of SPI1 (PU.1), RUNX1 and GATA2 (47, 48), which are known to be coopted for pathogenesis in AML. In addition, various transcription factors (e.g. RUNX1, CBFβ or RARα) are involved in genetic alterations that form aberrant fusion proteins in AML (47, 49). These fusion proteins often perturb the normal functions of these transcription factors and may also confer neomorphic activities contributing to aberrant differentiation (47, 49). Interestingly, the stage at which these fusion proteins cause myeloid maturation arrest appears to be directly dependent on the nature of the fusion protein (50). Furthermore, in addition to the fact that AML-LSCs show aberrant lineage-skewing as well as differentiation arrest, there is now emerging evidence to show that cells with a more differentiated phenotype can also regain the characteristics of AML-LSCs (51). The degree of plasticity that can be attained by blasts remains largely unexplored in AML, with open questions regarding potential cell-intrinsic and microenvironmental contributions (52). This ability of AML cells to de-differentiate into AML-LSCs may play a significant role in disease relapse and refractoriness to therapies (51, 53), contributing to the elusiveness of the LSC as a drug target.

In normal hematopoiesis, the transcriptional states of HSCs are defined by the gene networks that drive self-renewal, while lineage-specifying transcription factors exert their effects upon activation in downstream progenitor cells. Some of the self-renewal-associated genes and gene-networks that are active in HSCs are the clustered homeobox (HOX) genes, the three amino acid loop extension (TALE) domain proteins MEIS1 and PBX1 (54, 55), the transcription factor EVI1 (56), the WNT signaling pathway (57) and the RNA binding proteins MUSASHI2 (58, 59), STAUFEN2 (60) and SYNCRIP (61), to name a few. Not surprisingly, studies have shown that many of these gene-networks are aberrantly activated in AML, with a concomitant dysregulation of the expression or activity of lineage-specifying transcription factors (62). Furthermore, these signatures are highly predictive of chemotherapy response, and different LSC gene subset “scores” have been developed for clinical prognosis (63–65)

The transcriptional control of these “stemness” gene networks is tightly regulated by several distinct chromatin regulators in normal HSCs. These chromatin regulators maintain the balance between self-renewal and differentiation in normal HSCs (6). These regulators include the mixed-lineage leukemia gene (MLL) (66, 67), the histone methyltransferase DOT1L (68), the histone acetyl-transferases KAT6A (MOZ) (69), KAT8 (MOF) (70, 71) and KAT7 (HBO1) (72). Similarly, DNA methylation plays a crucial role in the control of HSC stem cell fate decisions. The DNA methyl-transferase DNMT3A regulates the expression of stem cell developmental programs, such as the clustered *HOX* genes (73, 74). Mutations in DNMT3A are frequently found in both pre-leukemic as well as leukemic states and are correlated with poor prognostic outcomes (75–77). Other epigenetic regulators such as TET2 (78), ASXL1 (79), and splicing regulators such as SRSF2 (80) and SF3B1 (81, 82) are also commonly involved in regulating the normal stem cell state. Large-scale sequencing studies have found that these epigenetic and transcriptional regulators constitute one of the most common class of genes mutated in AML (83, 84). Interestingly, mutations in these classes of genes are early events in AML pathogenesis and it is now believed that these mutations set the stage for the establishment of a pre-leukemic state.

Given their established importance in maintaining the LSC-state, drugs targeting chromatin-modifying proteins are being actively pursued in the clinic. One of the most promising targets for AML in this class is the histone methyltransferase DOT1L whose deletion or pharmacological inhibition shows potent effects in multiple preclinical models of AML (68, 85). However, phase I/II clinical trials with the DOT1L inhibitor pinometostat showed limited efficacy, due to pharmacological limitations of the drug (86). Since DOT1L is a highly selective regulator of the AML oncotranscriptome in several AML subtypes, such as KMT2A, MLLT10 or NUP98-rearranged AML, as well as of NPM1 mutant AML, further investigation of DOT1L inhibition with better pharmacological properties (85, 87) is warranted. Another promising epigenetic regulator in this leukemia subtype setting is Menin. Drugs targeting the interaction of Menin with KMT2A are being tested in separate clinical trials (NCT05153330, NCT0498855, NCT04067336, NCT05360160, NCT04811560, NCT05326516, NCT04065399). Recently, results from the AUGMENT-101 clinical trial with the menin inhibitor revumenib revealed a 53% overall response rate, with 30% of the patients showing complete remissions (88). Given that these were relapsed/refractory AML patients who had failed multiple previous lines of therapy, these results are remarkable, and highlight the potential of chromatin-targeting therapies in AML. Another clinical trial (KOMET-101) (89) with the structurally distinct MLL-Menin inhibitor Ziftomenib is currently ongoing and results are awaited. Clinical testing with inhibitors of other epigenetic regulators that are either mutated or recognized as non-oncogene dependencies in AML are also ongoing, including PRMT5, LSD1 and BRD4 inhibitors, although their roles in AML LSC-regulation is unclear (90).

### Metabolic states

It has recently been appreciated that several metabolic features distinguish AML-LSCs from HSCs. Studies using cellular efflux of the dye Hoechst 33342 as a phenotypic strategy for marking stem cells showed that this dye efflux capacity is altered in AML-LSCs, allowing their separation from the leukemic bulk (91, 92). These so-called side-population cells could enrich the LSC fraction, indicating that similar to normal HSCs, AML-LSCs are metabolically active in transporting this dye due to action of the G2 multidrug transporter (93, 94). Furthermore, it was shown that LSCs display higher levels of mitochondrial oxidative and lipid metabolism compared to glycolysis-driven quiescent HSCs (95). A recognized hallmark of cancer cells is their reliance on glycolytic metabolism, termed the Warburg effect. Indeed, it has been shown to be the preferred metabolic route for the bulk population of many tumor types. However, cancer stem cells from various solid and hematological cancers rather depend on oxidative phosphorylation (OXPHOS) for their survival (96). LSCs activate mitochondrial metabolism to coordinate regeneration and are unable to utilize glycolysis effectively. This vulnerability has thus been exploited in the development of targeted inhibitors that create synthetic lethalities in LSCs only, but not HSCs. For example, the BCL-2 inhibitor venetoclax has been paired with heme biosynthesis and amino-acid catabolism inhibition in proof-of-principle preclinical studies in AML (97, 98). The mechanism by which cancer cells regulate the balance between OXPHOS and glycolytic metabolic pathways is not yet fully understood, but recent studies have pointed to oncogene-induced “waves” of gene regulation that enable switching between the two states (99).

Importantly, OXPHOS inhibitors have shown efficacy in AML and are currently being tested in clinical trials (100–103). Another metabolic therapeutic window for targeting AML-LSCs relates to amino acid and lipid utilization. Notably, it was demonstrated that AML-LSCs from newly diagnosed samples upregulate amino acid metabolism for their survival and that pharmacological inhibition of this pathway could selectively eradicate AML LSCs (98). It was also found that compared to new diagnoses, LSCs from relapsed AML samples were instead dependent on increased fatty acid metabolism and may therefore require different therapeutic strategies (98).

An alternative strategy for targeting mitochondrial function involves the inhibition of Dihydroorotate dehydrogenase (DHODH), a flavoprotein located in the inner mitochondrial membrane. DHODH is interesting because it connects two important nodes recognized to be important in AML. It plays a crucial role in *de novo* pyrimidine synthesis, connecting nucleotide production with energy metabolism and the generation of reactive oxygen species (ROS). Recent studies have demonstrated that blocking DHODH activity, resulting in pyrimidine deprivation, induces the differentiation or death of AML blasts (104–106). It was further demonstrated that chemotherapy-resistant cells rely on pyrimidine synthesis and that combining DHODH inhibition with standard chemotherapy significantly reduces tumor burden *in vivo* (107). A recent study showed that a novel DHODH inhibitor AG636 induced potent anti-AML as well as anti-LSC effects with limited effects on normal hematopoiesis in preclinical model of MLL-AF9 AML. Mechanistic analysis demonstrated that DHODH inhibition led to the reduction of protein synthesis rates, which is a known metabolic vulnerability of AML-LSCs (108). Based on a number of promising preclinical studies, clinical trials on DHODH inhibition were initiated to leverage this selective vulnerability in AML cells, but at least two of these trials BAY2402234 (NCT03404726 – terminated due to limited efficacy) or with PTC299 (NCT03761069) have been discontinued. Thus, clinical translation of this highly promising metabolic vulnerability with LSC targeting potential remains to be realized.

Aside from the metabolic changes seen in AML-LSC regardless of AML mutational subtypes, more specific metabolic alterations are seen in AML subtypes that bear mutations in specific metabolic regulators, such as isocitrate dehydrogenases (IDH1/2) or TET2 (109). IDH1/2 mutations result in the production of 2-hydroxyglutarate (2-HG), an oncometabolite that disrupts epigenetic regulation and drives malignant hematopoiesis (110). 2-HG is an inhibitor of TET2, a dioxygenase enzyme that enables DNA promoter demethylation (111, 112) and is also mutated in AML, being mutually exclusive with IDH1/2 mutations. The accumulation of 2-HG due to IDH1/2 mutations in AML has been linked to the accumulation of reactive oxygen species (ROS) and the depletion of cellular NADPH, thus creating distinct, IDH mutation-initiated metabolic states. It remains to be known whether these changes are selective to LSCs and how these metabolic alterations can be therapeutically exploited for LSC-targeted therapies. In the context of leukemia with TET2 mutations, it has been recently shown that supplementation with Vitamin C - a co-factor of the TET2 enzyme - can rescue the function of wildtype TET2 and restore normal DNA methylation. Following successful clinical validation, this strategy may potentially provide a therapeutic avenue for the 30-50% of AML patients that harbor TET2 pathway disruptions (109, 113). These proof-of-principle studies demonstrate how metabolic vulnerabilities of AML-LSCs in general or those created by specific mutations in AML can be exploited for therapy (114).

### The role of the BM niche in the emergence of AML

HSCs reside in the bone marrow niche, a specialized environment that protects their quiescent state. Cell cycle quiescence allows for the limitation of oxidative stress from mitochondrial respiration and avoiding exhaustion from excessive cell cycling and proliferation (115–117). The hypoxic conditions in the bone marrow nice result in the stabilization of the hypoxia-inducible factor 1 (HIF-1), a transcription factor that promotes the expression of glycolytic genes (118). The depletion of HIF-1α, a monomer of the HIF-1 heterodimer formed with HIF-1β, leads to an increase in ROS and OXPHOS and ultimately, loss of quiescence (119).

The hypoxic state of the bone marrow niche also contributes to the therapy resistance features of LSCs (120, 121). A recent study found that AML cells that persisted after chemotherapy treatment used glutamine for pyrimidine and glutathione generation. Malignant pyrimidine synthesis also required aspartate, which was provided by stromal cells in the niche and drove the metabolic adaptation of residual AML cells (107). Furthermore, stromal cells have been found to provide mitochondria to AML cells, supporting their energy production (122–124). The adipocyte niche within the bone marrow has also shown to be protective for LSCs (125, 126), along with the adhesion molecule and cytokine environment in the bone marrow niche, reviewed in (127). Indeed, clinical trials with the E-selectin ligand inhibitor uproleselan (NCT03616470), which blocks the interaction of AML blasts and LSCs with the bone marrow vasculature, are underway for AML (128).

## LSCs as prognostic biomarkers

### Identifying pre-leukemic stem cells: lessons from clonal hematopoiesis

As mentioned previously, early studies on normal and malignant hematopoiesis relied on the characterization of cell-surface markers by flow cytometry and limiting dilution experiments to determine long-term reconstitution of the entire heterogeneity of normal or leukemic hematopoiesis in mouse models. More recently, emerging techniques including next-generation sequencing (NGS) and the computational deconvolution of complex clonal hierarchies have allowed new insights into the hematopoietic system. Among these is the understanding of age-related clonal hematopoiesis (CH), a phenomenon in which the progeny of certain HSC clones expands progressively, due to selective advantage conferred by accumulated mutations in the HSC (reviewed in (129, 130).

CH holds the potential to predispose to AML, a genetically heterogenous disease where typically patients harbor several co-occurring mutations (109). It is now becoming apparent that few mutations in a small number of genes such as DNMT3A, TET2 and TP53, among others, are present in long-lived self-renewing cells, while other mutations are acquired as subsequent hits, in a sequential, multi-step process (129). Interestingly, the first few mutations are more likely to expand the pre-leukemic stem cell pool, leading to the clonal expansion of these mutant HSCs and in consequence, CH. Subsequently, the acquisition of mutations in genes that block differentiation may lead to the establishment of overt leukemia. Thus, this step-wise process, confers clonal heterogeneity and distinguishes pre-leukemic clonal populations which can eventually lead to the establishment of overt AML (131). With this knowledge, NGS-based studies are now able to investigate the impact of anti-AML therapies on these distinct leukemic clones, thus shedding new light on the response of patients to targeted treatments (132, 133). Further, the assessment of CH and AML genotypes have changed clinical management to accommodate the evolving understanding of the biological complexity of the leukemic clone (134–136). CH remains under intense investigation and is beyond the scope of this review, but it serves to underscore the importance of charting a potential premalignant state for therapeutic translation in AML (137).

### LSC features and disease progression and relapse

A body of evidence supports that LSCs may drive chemotherapy resistance and thus disease relapse in AML (63, 120, 138–142). Using functional approaches, it has been shown that high LSC frequency at diagnosis is associated with enhanced engraftment in immunocompromised mice, high measurable residual disease load after chemotherapy, and poor survival outcomes in patients (143, 144). With greater insights from large sequencing efforts, an LSC expression signature was defined across patients and its correlation to adverse outcomes in AML patients was established (145). Indeed, in a study comparing prognostic factors, LSC frequency was found to be the strongest predictor of overall survival compared to other parameters, such as age, blast counts and genetic aberrations (146). It needs to be mentioned that while there is a body of functional and clinical evidence supporting the role of LSCs in chemoresistance and relapse, one recent study reported that LSCs may not be always resistant to treatment (147). Nevertheless, the eradication of LSCs is currently a therapeutic development goal for a wide variety of AML patients that fail to respond to currently-used therapies.

A greater understanding of the disease risk based on therapy response has led to recent revisions and updates of major treatment consensus guidelines. AML remains a disease with poor long-term survival rates, as most patients relapse despite achieving remission (148) (**Figure 1D**). Recently, the European LeukemiaNet revised both its AML clinical guidelines (in 2022), and its measurable residual disease (MRD) testing guidelines for AML (in 2021). The World Health Organization and International Consensus Classification for myeloid neoplasms were also updated and published in 2022 (149). As discussed in this review, LSCs have a preeminent role in relapse given their persistence after chemotherapy. Mechanistically, this was thought to be due to mutations that produce drug resistance, arising as a consequence of the mutagenic properties of chemotherapeutics (150). However, other lines of evidence have pointed to the pre-existence of drug-resistant cells (151). Deep-sequencing studies of paired samples of human AML at diagnosis and relapse have shown that relapse may arise from minor genetic sub-clones present at diagnosis that survive chemotherapy (151–153). The generation of resistant cells could thus occur before treatment (151), and selection would be driven by therapy (16, 18, 154). A recent study (155) combined genetic and functional analysis of sorted subpopulations and xenografts from initial diagnosis and relapse patient samples to resolve the cell types that are fated to drive relapse. The findings elucidated two major patterns of relapse. In the first, relapse originated from a rare LSC population with a hematopoietic stem/progenitor cell phenotype, already present at diagnosis before therapy initiation. In the other model, relapse arose from larger sub-clones of immunophenotypically committed leukemia cells that retained strong transcriptional signatures of stemness (155). These data showed that AML undergoes complex clonal evolution in the pre-leukemic and LSC compartments, supporting the concept that instead of the emergence of clones with new mutations due to chemotherapy, the selection pressure of anti-AML therapies may select for resistant clones due to the dormancy or epigenetic plasticity of these clones (6). These and other recent reports reinforce the notion of the LSC as “a moving target” (156) – a cell state that may exist prior to therapy or can emerge from other clones that take on stem cell characteristics following treatment.

Therefore, the identification of pre-existing LSCs as well as of pre-leukemic clones that can acquire LSC characteristics may help improve methods for disease management and monitoring in AML, although it remains challenging. Dispersed residual leukemic cells (1 in 10^4^ to 10^6^ white blood cells at clinical remission (157)), including LSCs, may fall below the limit of detection of measurable residual disease (MRD) by multicolor flow cytometry (MFC) or molecular quantification by real-time quantitative PCR, the most widespread methods for detection (158, 159). MRD can also be measured by NGS and other methods with high sensitivity, including droplet-digital (dd) PCR (160). The technology used to assess MRD is critical and the standards for its interpretation across different subtypes and disease stages remain under discussion in multiple specialist working groups (reviewed in (161)). The mounting impact of MRD assessment on treatment decision-making remains to be standardized (162) and highlights the relevance of LSCs and the difficulty posed by the molecular heterogeneity of AML.

Since relapse may arise from genetically diverse dormant populations that can take on LSC properties, therapeutic approaches that target only the features of the dominant clone would prove ineffective. Therefore, any anti-LSC therapies would have to comprehensively address these recently appreciated features of LSC biology in order to achieve long-lasting cures in AML.

## Concluding remarks

### Challenges and open questions regarding targeting LSCs

Research over the last two decades has enabled a better understanding of the concept of LSCs, their importance on AML progression, pathogenesis, and disease refractoriness. The main challenge in the near future is to determine how this rapidly growing information can be harnessed to develop effective LSC-targeting therapies. To this end, it is critical to expand on the search for clinically actionable LSC-specific targets. Rapid strides have been made in this direction as mentioned previously in this review. Immunophenotypic markers, including CD96 (20, 163), CD123 (164), CD47 (165), and TIM-3 (166), expressed on pre-leukemic and leukemic LSCs, are being explored as potential targets for immunotherapy. Unique metabolic features that distinguish AML-LSCs from HSCs are also being exploited as a therapeutic vulnerability in AML. It is already apparent that drugs such as venetoclax may be targeting the pro-survival mechanisms that are active in AML-LSCs, which may partly explain their efficacy in AML (114). One of the long-standing challenges in the AML-LSC field is this: how can we reverse transcriptional “stemness” networks that are constitutively activated in AML and drive limitless self-renewal of AML-LSCs? One way of addressing this important problem is by targeting epigenetic regulators that sustain the expression of genes important to stemness. Several studies have identified upstream epigenetic regulators of AML-LSC genes, and the clinical translation of these findings has brought mixed results. For example, an inhibitor of the histone methyltransferase DOT1L which reverses *HOX/MEIS* activation in AML was found to have modest efficacy in a clinical trial for patients with MLL-rearranged AML. The efficacy of other epigenetic regulators that can control the transcription of self-renewal associated genes in AML, such as inhibitors of the MLL-Menin interaction is promising (88, 167–172). Recent approaches for the high-throughput identification of LSC self-renewal regulators have combined whole-genome or targeted genetic perturbation platforms and single-cell RNA sequencing to gain deeper insights into HSC and AML-LSC self-renewal at the single-cell level (72, 173–175). These strategies will help nominate several novel candidates for bench-to-bedside translation of anti-AML-LSC therapies. Once again, the time has come to revisit self-renewal in AML – a concept that has presented both challenges and opportunities in the advancement of therapies targeting the elusive cancer stem cell.

## Author contributions

KB and AD conceived, wrote and revised the manuscript. All authors contributed to the article and approved the submitted version.
